# Alzheimer’s Disease, Diagnosis and the Need for Biomarkers

**DOI:** 10.4137/bmi.s682

**Published:** 2008-05-27

**Authors:** Claudie Hooper, Simon Lovestone, Ricardo Sainz-Fuertes

**Affiliations:** King’s College London, MRC Centre for Neurodegenerative Research, Institute of Psychiatry, De Crespigny Park, Denmark Hill, London, SE5 8AF, U.K

**Keywords:** biomarker, Alzheimer’s disease, tau, β-amyloid, memory, neuro-imaging, psycho-metric testing

## Abstract

Alzheimer’s disease (AD) is a progressive neurodegenerative disorder of aging that presents with memory loss, disorientation, confusion and a reduction in cognitive ability. Although a definite diagnosis of the disorder can only be made post-mortem by histopathological analysis, a number of methods are currently available for the *in vivo* assessment of AD including psycho-metric tests and neuro-imaging. However, these clinical assessments are relatively nonspecific and imaging is very costly. Genetic testing can be performed if familial AD is suspected, although such cases represent a very small minority of total AD cases. Apolipoprotein E genotype provides a measure for analysing the risk of developing AD, but does not act as an absolute predictive biomarker for AD. Therefore there is a need for an accurate, universal, specific and cost-effective biomarker to facilitate not only ante-mortem diagnosis of AD, but also to allow progression of the disease and response to therapy to be monitored. This is the ultimate goal that our group is pursuing through the pan-European AddNeuroMed project.

## Introduction

Definite diagnosis of Alzheimer’s disease (AD) can only be made upon post-mortem examination of brain tissue for evidence of the presence of abnormal protein structures know as neurofibrillary tangles (NFTs) and senile plaques in the hippocampus and cortex of afflicted individuals (Mattson, 2004;  Braak and Braak, 1998; Dickson, 1997; Agronin, 2008). However, a number of ante-mortem diagnostic tests for AD are becoming available and are being developed with the aim of enabling the trained clinician to make a fairly accurate diagnosis of AD in conjunction with information obtained from a comprehensive clinical interview and detailed family history. Such tests are the subject of this review and include genetic profiling, psycho-metric testing, neuro-imaging and the assessment of protein or metabolic products in biological fluids. Nevertheless, each test comes with its own set of inherent problems such as limited disease specificity, high expense or high labour demands (discussed below). Thus, there is a need to develop new accurate, inexpensive, easy to perform tests for ‘accessible’ biomarkers of AD (unlike NFTs and senile plaques that are inaccessibly located as insoluble aggregates in the brain) to enable diagnosis. This need is especially pressing considering that AD is becoming more prevalent as life expectancy has increased due to improvements in healthcare and lifestyle. The AddNeuroMed project, which our group is intimately involved in aims to address this and represents the largest European effort to identify biomarkers of AD for the use in trials (Lovestone et al. 2007; www.innomed-addneuromed.com).

AddNeuroMed is a consortium of clinicians and researchers in the field of AD from across the European Union with study sites in Finland, France, Greece, Italy, Poland and the UK. Biological samples, including blood, CSF, and urine are being collected at these sites from a large cohort of elderly European volunteers with either AD, mild cognitive impairment (MCI: transitional state between normal cognition and AD) or normal memory function with the intention of gathering genetic, transcriptomic, proteomic and lipidomic data. The participants have also agreed to neuro-imaging procedures to provide a more in depth assessment of their clinical phenotype. Having begun in 2006, the AddNeuroMed study is still in its infancy with longitudinal follow-ups still in progress for the majority of participants, which will ultimately enable the assessment of disease progression. In parallel to clinical data collection, pre-clinical studies are being conducted using tissue culture, drosophila and mouse models of AD to screen for potential biomarkers and putative signaling pathways involved in disease pathogenesis. The AddNeuroMed study also serves as a pilot project for the Innovative Medicines Initiative, which is a European consortium aimed at improving pharmaceutical research communication and collaboration with clinical and research groups across Europe.

## Alzheimer’s Disease

AD is a progressive, dementing (from the Latin meaning ‘without a mind’), neurodegenerative disorder characterised by neuropathological signs and identifiable clinically by an insidious onset and deterioration of multiple higher cortical functions resulting in memory loss, disorientation, confusion, impaired concentration, alterations of language, learning and judgement and a widespread decline in general cognitive function (Tanzi and Bertram, 2005; Agronin). The defining classical hallmarks of AD are senile plaques and NFTs, which are concentrated predominantly in the hippocampus and the cortex (Goedert and Spillantini, 2006; Hardy, 2006). NFTs are composed of paired helical filaments (PHF) of an abnormal form of tau, an axonal microtubule associated protein (MAP) (Goedert, 1998). Normally, tau is involved in tubulin polymerization and microtubule stabilization and is phosphorylated at two to three sites. In AD, tau becomes hyper-phosphorylated and consequently dissociates from microtubules and aggregates into PHFs forming NFTs. The reduced binding of phosphorylated tau to microtubules results in the impairment of vital cellular processes such as axonal transport and ultimately culminates in the degeneration of affected neurones.

Senile plaques mainly consist of extracellular deposits of β-amyloid (Aβ), a peptide produced from the proteolytic cleavage of β-amyloid precursor protein (APP). APP is subject to proteolytic processing at three sites by the action of α, β and γ secretase. Cleavage by β and γ secretase releases Aβ, whereas α cleavage is harmless. Intense efforts have been directed towards the identification of α, β, and γ secretase. ADAM 10 (Lammich et al. 1999) and tumor necrosis factor alpha converting enzyme (Buxbaum et al. 1998) have been reported to mediate α-cleavage of APP. The aspartyl protease termed BACE for beta-site APP-cleaving enzyme has been demonstrated to mediate β cleavage (Vassar et al. 1999) and a multi-protein complex consisting of presenilin-1 (PS-1) (Wolfe et al. 1999), nicastrin (Li et al. 2003), Aph-1 and Pen-2 (Kimberly et al. 2003) is thought to be involved in γ cleavage (De, 2003).

There is no question that AD pathology is characterised by the presence of senile plaques, NFTs and neuronal loss, but the precise sequence of events underlying the pathogenesis of AD remains unknown. Probably the most influential theory to date has been the amyloid cascade hypothesis (Hardy and Higgins, 1992; Hardy, 2006). According to this theory APP synthesis and or processing become abnormal due to a combination of genetic and environmental factors, which results in the formation of senile plaques. The presence of plaques subsequently exerts a deleterious effect on neuronal survival, leading to the development of PHFs and NFTs. Over time the gradual accumulation of these histopathological features is believed to cause neuronal death and neurotransmitter deficits ultimately culminating in dementia.

## What is a Biomarker?

A biomarker is defined as a biological parameter that can be objectively measured and evaluated as an indicator of normal or pathological states or pharmacological responses to therapeutic intervention (Atkinson et al. 2001; Lovestone et al. 2007b; Lovestone, 2006). A biomarker could therefore be used for disease diagnosis, as a predictive marker of disease progression and prognosis or as a tool for staging a condition or monitoring a response to targeted treatment. An ideal biomarker of AD would therefore encompass all of these parameters.

A classical biomarker can be a measure of function of a tissue or organ that is detected using clinical examination. Examples of such biomarkers include measurements of heart rate or changes on an electrocardiogram (ECG), temperature or blood glucose levels to name but a few. Imaging techniques including X-rays, computed tomography (CT), magnetic resonance imaging (MRI) or positron emission tomography (PET) can also be used to provide a measure of the function of a tissue or organ. A molecular biomarker can be a genetic trait, a biochemical change in protein expression or protein activity or an alteration in metabolite levels reflecting anomalous activity of a certain biochemical pathway relating to health or indeed disease. Tests for biomarkers must demonstrate high accuracy, specificity and inter-assessment test-retest reliability if they are to be used with success and confidence in the clinical setting.

A biomarker, classical or molecular, may also be used as a ‘surrogate endpoint’ to substitute for a ‘clinical endpoint’ (Atkinson et al. 2001; Lovestone et al. 2007b; Lovestone, 2006). A ‘clinical endpoint’ is an outcome that is measured during a clinical trial. A clinical endpoint is a characteristic or variable that informs how the patient feels, functions or survives providing a measure of morbidity or mortality. Therefore, surrogate endpoints can be used to evaluate safety and clinical benefit and to predict the effect of a therapeutic intervention in a shorter timeframe. Only a small minority of biomarkers become established surrogate endpoints for a particular disorder. Blood pressure is an example of a surrogate endpoint for cardiovascular disease, confirmed by the vast amount of epidemiological evidence (Desai et al. 2006).

## Genetic Traits and Susceptibility Loci Associated with AD

Early onset forms of Familial Alzheimer’s disease (FAD) typically present before the age of 65 and as early as 30 years of age and have been linked to mutations in APP, PS-1 and PS-2 (Van Broeckhoven, 1995). These mutations adversely affect APP processing and result in the increased production of insoluble Aβ, which is deposited in the form of senile plaques. Note PS-2 can substitute for PS-1 in the γ secretase complex (Steiner et al. 1999). Genetic screening, therefore, provides an accurate diagnosis of AD in the small number of familial cases (approximately 2–7% of total cases).

Sporadic AD is a polygenic disease that accounts for the majority of AD cases and exhibits a later age of onset in comparison to FAD, typically affecting individuals over 65 years of age. The presence of the apolipoprotein E (apoE) ɛ4 allele is a risk factor for sporadic AD and increases the chance of developing the condition depending on the number of copies of this allele an individual harbours (Saunders et al. 1993; Yoshizawa et al. 1994). The presence of this allele, however, is by no means a definitive determinant for the development of AD. The apoE genetype alone, therefore, cannot be relied upon as a robust predictive diagnostic biomarker for AD and is best described as a trait as opposed to a state marker of AD.

## Assessment of Cognitive Function and Memory as a Biomarker of AD

The diagnosis of AD is based on either the National Institute of Neurological and Communicative Disorders and Stroke—Alzheimer’s Disease and Related Disorders Association (NINCDS-ADRDA) criteria or on the standards set out in the Diagnostic and Statistical Manual of Mental Disorders (DSM—the most up to date being the fourth edition test revision). Diagnostic criteria set out in these documents include the development of multiple cognitive deficits manifesting in memory impairment and one or more cognitive disturbances such as aphasia, apraxia, agnosia, or disturbances in executive functioning and activities of daily living. Cognitive deficits must exhibit gradual onset, cause impairment in social or occupational functioning and the presence of other CNS or systemic conditions known to cause dementia must be excluded (Thomas, 2008; Agronin, 2008).

Many psycho-metric examinations are available as tools for the diagnosis of AD and provide an assessment of the key defects of cognitive function and memory associated with AD set out by the DSM and NINCDS-ADRDA working standards. These tests also can be used to stage disease progression allowing the assignment of disease severity (Rush et al. 2000; Burns et al. 2004; Behl et al. 2005; Harvan and Cotter, 2006). The advantages of such AD tests are that they are inexpensive, do not require extensive training to be administered and they exhibit good test-retest reliability. Psycho-metric tests for AD include the mini-mental state examination (MMSE), the functional assessment staging (FAST), the Alzheimer’s disease assessment scale cognitive subscale (ADAS-Cog), the clock drawing test, the severe impairment battery (SIB), the modified Alzheimer’s disease cooperative study—activities of daily living (ADCS-ADL), the behavioural rating scale for geriatric patients (BGP), the neuropsychiatric inventory (NPI) and the clinicians interview-based impression of change plus caregiver input (CIBIC-Plus) (Rush et al. 2000; Burns et al. 2004; Behl et al. 2005; Harvan and Cotter, 2006). These tests involve a series of questions and tasks for the patient to perform, which the interviewer uses to explore registration (the ability to recognise and name specific items), encoding (the processing and combining of new information), concentration, memory, language skills and the ability to perform daily living tasks. Psycho-metric tests are useful for quantifying the degree of cognitive impairment, monitoring disease progression and planning adequate care for dementia patients, but they do not discriminate AD from other types of dementia and the tests are time consuming to perform. Moreover, some psycho-metric tests are culturally bound and their results may be affected by differences in ethnicity and education level leading to false positive results.

## Neuro-imaging as a Biomarker of AD

Neuro-imaging techniques can be employed as a tool for the diagnosis of AD and using these techniques the presence of vascular damage can also be assessed, which is helpful for the discrimination between vascular dementia and AD (Agronin, 2008). The main disadvantage associated with neuro-imaging is that it is expensive to perform routinely and the scans are distressing for demented subjects. Volumetric magnetic resonance imaging (vMRI) measures brain volume to provide an indication of neurodegeneration. At a regional level, a reduction in the size of the hippocampus (Kaye et al. 1997) and the entorhinal cortex (the input to the hippocampus) is apparent in AD (Du et al. 2003), whilst at the level of the whole brain, a reduction in total size accompanied with ventricular enlargement is evident (Silbert et al. 2003; Fox et al. 2000). Magnetic resonance spectroscopy is another form of neuro-imaging, which demonstrates a reduction in the concentration of N-acetyl aspartate (a putative neuronal marker) in AD brain compared to healthy controls (Meyerhoff et al. 1994; Frederick et al. 2004).

Functional imaging using [18]fluoro-deoxy-glucose (FDG) PET is another neuro-imaging tool available for the assessment of AD. PET provides a measure of glucose utilization and such imaging reveals a reduction in the metabolic rate of glucose in AD in the posterior cingulate, the parietal, the temporal and the prefrontal cortices (Alexander et al. 2002). PET imaging studies using radio-ligands such as the Pittsburgh Compound B (PiB) that bind Aβ can also be used as a diagnostic measure for AD (Klunk et al. 2004; Nordberg, 2007) and dementia with Lewy bodies (DLB), a disease that also features amyloid plaques (Rowe et al. 2007). Using PiB, or similar amyloid tracers, the efficacy of anti-amyloid therapies can be monitored as a means to reduce amyloid plaque burden and perhaps improve cognitive function *in vivo.* Interestingly, PiB has also been shown to bind to NFTs as well as Aβ (Lockhart et al. 2007), thus, in the future perhaps enabling clinicians to perform virtual brain biopsies (Agronin, 2008).

## Putative Proteinaceous and Metabolic Biomarkers of AD

The cerebrospinal fluid (CSF) bathes the brain and spinal cord and therefore this fluid might reflect pathological changes occurring in the central nervous system (CNS), which relate to neurodegen-erative disorders. In AD, CSF levels of total tau are elevated (Vandermeeren et al. 1993; Blennow et al. 1995; Vigo-Pelfrey et al. 1995), although total tau is also increased in the CSF in vascular dementia (Andreasen et al. 1998; Nagga et al. 2002) and in fronto-temporal dementia (Green et al. 1999). Elevated levels of phosphorylated-tau are also apparent in AD and in contrast to total tau this marker of disease seems to be more specific to AD as opposed to other dementias (Vanmechelen et al. 2000; Itoh et al. 2001; Parnetti et al. 2001; Nagga et al. 2002). Reduced levels of Aβ1–42 provide another CSF-based biomarker of AD. However, Aβ1–42 is also decreased in other dementias and is therefore a relatively non-specific marker of AD (Nagga et al. 2002; Sjogren et al. 2000). Together the combined analysis of CSF total tau and Aβ1–42 levels provides the most sensitive and specific laboratory-based test for AD and MCI (Andreasen et al. 2001; Hulstaert et al. 1999) that in conjunction with neuro-imaging and a detailed case history could be effectively implemented in the clinic and to date provides the gold standard to which all other tests for putative molecular biomarkers of AD should be compared.

As an alternative to CSF, peripheral blood provides a readily available plentiful source for laboratory testing. The main advantage of using blood over CSF as a source for biomarker assessment is that it can be easily attained from patients and it avoids the need for a lumbar puncture, which is a relatively unpleasant invasive procedure and potentially dangerous to perform in demented patients. There is a growing body of evidence to suggest that blood brain barrier (BBB) integrity is compromised in disease states including AD (Hawkins and Davis, 2005; Persidsky et al. 2006) and it is possible that central metabolic changes associated with disease might also be anomalous in the periphery; as such the blood might reflect CNS status.

Numerous blood based biomarkers of AD have been described in the literature, but as yet none of these biomarkers have been sufficiently validated for routine use in the clinic. We have demonstrated that the enzyme glycogen synthase kinase 3 (GSK3) is up-regulated in circulating peripheral white blood cells in AD (Hye et al. 2005). Furthermore, Aβ1–42 is up-regulated in blood plasma in individuals with familial mutations in APP, PS-1 or PS-2 (Scheuner et al. 1996), but Aβ1–42 is not elevated in sporadic cases of AD, therefore this state biomarker is of limited use in the general population. A plethora of other soluble blood-based biomarkers of AD have been described and include isoprostanes (lipid oxidation products) (Pratico et al. 2000), homocysteine (metabolic intermediate) (Seshadri et al. 2002), p97 (iron transport protein) (Feldman et al. 2001), interleukin 1, interleukin 6 (cytokines), α-1 antichymotrypsin (acute phase protein/protease inhibitior) (Licastro et al. 2000), α-2-macroglobulin (acute phase protein/protease inhibitor) and complement factor H (Hye et al. 2006).

Our group has employed image analysis of the entire plasma proteome using Two-dimensional-gel-electrophoresis (Hye et al. 2006). This technique enables the prediction of AD from control patients through pattern recognition with relatively high specificity and circumvents the issues arising from analysis of a single biomarker as a measure of disease status; such as poor reliability and specificity. An ELISA-based approach has also recently been used to identify 18 plasma proteins, including tumor necrosis factor alpha, epidermal growth factor, interleukin 11, interleukin 3 and TRAIL-R4 that can be used to accurately distinguish AD from control patients. Furthermore, this method enables the identification of patients that will progress to develop AD from a state of MCI (Ray et al. 2007). Thus, we believe that proteomic or indeed transcriptomic or metabolomic approaches should be used in the future to explore candidate biomarkers of disease. Such ‘ome’ wide profiling should provide a more specific and sensitive measure of disease, enabling signature panels of changing proteins, transcripts and/or metabolites to be identified and used as biomarkers or even clinical endpoints after rigorous validation.

## Concluding Remarks

There is a growing requirement for a clinical biomarker, or a set of biomarkers, of AD to enable accurate ante-mortem diagnosis of sporadic AD in a clinical setting without the need for laborious and non-specific psycho-metric testing or costly neuro-imaging technologies. Ideally a biomarker/or a panel of biomarkers for AD would allow the early prodromal diagnosis of AD and the biomarker(s) would correlate with disease progression and therapy. This would allow for the administration of drugs to patients before the onset of the core irreversible debilitating symptoms of AD, which is when treatment has been shown to exert the greatest clinical benefit. Furthermore, such a biomarker(s) would enable the effectiveness of a given treatment to be readily tracked and assessed, which would allow for the best possible treatment regimens to be prescribed and tailored for an individual patient. Identification of such a biomarker or indeed panel of bio-markers is the main aim of the AddNeuroMed project and hopefully this is an achievable goal in the not too distant future.
